# Effectiveness of Posterior Decompression and Internal Fixation in Emergency Management of Thoracolumbar Fractures Complicated by Spinal Cord Injury

**DOI:** 10.1155/emmi/7832479

**Published:** 2024-12-19

**Authors:** Jian Li, Tao Zhou, Sen Lin, Hongliang Wang

**Affiliations:** Department of Orthopedics, Ma'anshan People's Hospital, Ma'anshan 243000, China

**Keywords:** clinical effectiveness, internal fixation, posterior decompression, spinal cord injury, thoracolumbar fracture

## Abstract

**Objective:** This study evaluates the effectiveness and timeliness of posterior decompression and internal fixation in the emergency management of thoracolumbar fractures complicated by spinal cord injuries.

**Methods:** We retrospectively analyzed 40 patients treated at our hospital from January 2019 to February 2022. Each patient underwent posterior decompression and internal fixation, with preoperative and postoperative assessments including vertebral body height, American Spinal Injury Association (ASIA) score, Visual Analog Scale (VAS) score, and urodynamic indices.

**Results:** Postoperative improvements were noted in vertebral body height, with anterior and posterior heights increasing to 12.82 (± 1.23) mm and 3.21 (± 0.64) mm, respectively, and kyphosis angle improving to 14.26 (± 0.32). Significant enhancements were also observed in motor (from 40.78 [± 4.32] to 59.86 [± 1.37]) and sensory (from 45.98 [± 3.20] to 66.92 [± 1.28]) function scores, and a reduction in VAS score from 6.89 (± 0.78) to 1.78 (± 0.32). Urodynamic measurements showed increased maximum urine flow and detrusor pressure postintervention. All surgical wounds healed within two weeks without significant complications.

**Conclusion:** Posterior decompression and internal fixation significantly improve spinal stability, pain, motor, and sensory functions in patients with thoracolumbar fractures and spinal cord injuries, demonstrating its effectiveness and clinical utility.

## 1. Introduction

The thoracolumbar spine, characterized by the transition between thoracic kyphosis and lumbar lordosis, is particularly vulnerable to injury from external forces such as car accidents, violent impacts, and falls from significant heights [1–3]. Thoracolumbar fractures often result in severe clinical outcomes, including pain, neurological deficits, and even life-threatening conditions such as shock and loss of consciousness. These injuries frequently necessitate immediate medical attention in emergency settings to prevent long-term disability caused by unrelieved pressure on the cauda equina nerve and displaced bone fragments [4, 5].

Despite the high incidence of thoracolumbar fractures with spinal cord injury and their significant impact on patient health and quality of life, the optimal surgical intervention in emergency situations remains a subject of considerable debate. Current treatment typically involves surgical approaches such as decompression and internal fixation, which aim to relieve pressure and stabilize the spine [6]. However, the effectiveness of these interventions can vary significantly based on factors such as the exact nature of the injury, the patient's overall health, and the specific characteristics of the fracture [7].

In emergency settings, rapid and effective surgical intervention is crucial. Patients often present in critical condition, requiring swift assessment and treatment to minimize potential neurological damage and other complications. The primary goal of emergency surgery is to quickly stabilize the spine and decompress the spinal cord to improve patient outcomes.

Given these challenges, this study aims to fill a critical research gap by comparing the outcomes of anterior versus posterior decompression and internal fixation techniques in a controlled cohort. Our hypothesis posits that posterior decompression and internal fixation, due to its less invasive nature and direct approach to decompressing the affected spinal area, will result in better clinical outcomes regarding lower limb function, pain reduction, and overall recovery in patients with thoracolumbar fractures complicated by spinal cord injury.

The unique contribution of this research lies in its direct comparison of two prevalent surgical techniques, providing evidence-based insights that could guide clinical decision-making and improve patient prognoses. By focusing on a range of outcomes, including neurological recovery and functional improvement, this study focuses on the emergency surgical interventions following initial stabilization, examining the role of posterior decompression, and internal fixation in enhancing patient outcomes.

## 2. Data and Methods

### 2.1. General Data

To ensure the statistical validity of our study results, the sample size was carefully calculated. We aimed for a sample that would provide sufficient power to detect a clinically significant difference in outcomes between the two surgical approaches. Using G∗Power software for power analysis, we determined that a total of 40 patients would allow us to achieve an 80% power to detect a medium effect size (*f* = 0.25) with an alpha level of 0.05. This sample size calculation took into account the expected rates of recovery based on preliminary data and literature reviews, which suggest substantial variability in recovery outcomes among patients with thoracolumbar fractures. The chosen sample size is thus intended to balance the need for statistical power with the practical limitations often encountered in clinical research settings, where higher injury severity and specific inclusion criteria may limit participant availability.

A total of 40 patients with thoracolumbar fracture complicated by spinal cord injury were admitted to our hospital from January 2019 to February 2022 and treated with posterior decompression and internal fixation. The participants comprised 24 males and 16 females, categorized into three age groups: 27–35 years (10 patients), 36–50 years (15 patients), and 51–65 years (15 patients), with an average age of 52.78 (± 4.32) years. The injuries were primarily caused by car accidents (10 cases), falls from significant heights (15 cases), and violent injuries (15 cases). Most patients sustained injuries to the lumbar vertebrae (36 patients), while a smaller group had thoracic vertebrae injuries (4 patients). This study received ethical approval from the ethics committee of Ma'anshan People's Hospital (No: MH23156), and informed consent was obtained from all participants.

### 2.2. Diagnostic Criteria

All patients were diagnosed using a Siemens Somatom Definition AS 64-slice CT scanner, and GE Signa Pioneer 3T MRI. CT scans were performed with a slice thickness of 2 mm, at 120 kV and 200 mA. MRI settings included a T2-weighted sequence with a slice thickness of 3 mm.

### 2.3. Inclusion Criteria

The inclusion criteria include the following: (1) all patients were admitted to the hospital for surgical treatment 24 h after injury; (2) those who met the surgical indications; (3) those without chronic diseases such as hypertension and diabetes; (4) those who were well informed and signed informed consent.

### 2.4. Exclusion Criteria

Exclusion criteria included patients with poor compliance, coagulopathy, severe cardiopulmonary, renal, or hepatic impairment. Specific comorbidities considered for exclusion were systemic inflammatory diseases, autoimmune disorders, and any chronic condition potentially affecting surgical outcomes or postoperative recovery.

### 2.5. Surgical Methods

All patients underwent posterior decompression and internal fixation in a prone position under general anesthesia, which was specifically tailored to each patient's health status. Preoperative preparations were comprehensive: patients fasted for at least 12 h to ensure an empty stomach, which is crucial for safe anesthesia administration. Medication adjustments, particularly for those taking anticoagulants or anti-inflammatory drugs, were managed under the guidance of an anesthesiologist to minimize surgical bleeding and inflammation. To optimize surgical access and patient comfort, lumbar extension was facilitated by placing a specifically designed air cushion under the waist of the patient.

The surgical approach was meticulously planned and executed. The injured spinal segment was first identified through preoperative MRI scans and further confirmed intraoperatively with a C-arm X-ray machine to ensure precise localization. A longitudinal incision was then made directly over the identified segment to expose the injured lumbar spine. Special attention was given to the insertion of four pre-positioned pedicle screws, which were adjusted for strength and depth to ensure they were securely fixed at approximately 75% above the height of the vertebral laminae, thus stabilizing the spine adequately. X-ray guidance was continually employed to assess the extent of spinal stenosis and to direct the decompression process accurately.

The initial phase of the surgery focused on decompressing the spinal nerve tissue to relieve pressure effectively. This was followed by pedicle screw fixation, which aimed to secure vertebral alignment and maintain spinal integrity. In cases where bone fragments posed a threat to the dural sac—such as in three severely injured patients—these fragments were meticulously removed before proceeding with any necessary dural repair and final fixation, thereby preventing further neurological damage.

Postoperative care was rigorously applied to ensure optimal recovery. Patients were monitored closely for signs of infection, proper wound healing, and neurological improvement. Pain management was tailored to individual needs, and mobility was gradually reintroduced under the supervision of physiotherapists to prevent muscle atrophy and promote recovery. Regular follow-up appointments were scheduled to assess recovery progress and to make any necessary adjustments to their rehabilitation plan.

### 2.6. Observation Indexes

1. Make clear the surgery situation of patients, record the surgery time and the intraoperative blood loss by counting the dressings, check the admission and discharge time, and record the length of stay of patients.
2. Identify the height of the anterior border of the patient's vertebral body before and after the surgery.
3. After 30 days of intervention, Spinal Cord Injury Severity Score formulated by the American Spinal Injury Association (ASIA) was used to evaluate the lower limb motor function and sensory function of patients. With a full score of 100, the higher the score, the better the lower limb motor function and sensory function of patients.
4. After 30 days of intervention, Visual Analog Scale (VAS) was used to evaluate the pain of the patients before and after surgery. With a score range from 0 to 10, the higher the score, the more severe the pain of the patients.
5. Urodynamic indicators were used to evaluate the urodynamic status of the patients, including maximum urine flow, maximum systolic pressure of detrusor, and bladder compliance. All the above treatments need to be evaluated under the intervention of a urine collector or catheter.

### 2.7. Statistical Methods

With statistical software SPSS 20. 0 used for data analysis, the measurement data were represented by ($\bar{x}\pm s$), and *t*-test was used for comparison between the two groups. The count data are expressed by rate and verified by *x*^2^ test. The difference was statistically significant (*p* < 0.05).

## 3. Results

### 3.1. Comparison of Surgical Indicators

All the 40 patients included in this study received posterior decompression and internal fixation, with average surgery time of 115.34 (± 7.38) min, intraoperative blood loss of 312.98 (± 40.37) mL, and hospital stay of 16.83 (± 0.48) d.

### 3.2. Comparison of Anterior Border Height of Vertebral Body in Patients

The anterior border height of the injured vertebral body in patients treated with posterior decompression and internal fixation showed significant improvement. The anterior border height of the injured vertebral body was recovered to 12.82 (± 1.23) mm on average, the posterior border height of the injured vertebral body was recovered to 3.21 (± 0.64) mm on average, and the angle of spinal kyphosis was recovered to be 14.26 (± 0.32) on average.

### 3.3. Comparison of ASIA Score and VAS Score of Patients

The motor function score and sensory function score in the ASIA scale before surgery were 40.78 (± 4.32) and 45.98 (± 3.20), respectively, while those after surgery were 59.86 (± 1.37) and 66.92 (± 1.28). The VAS score of patients was 6.89 (± 0.78) before intervention, while that of patients after intervention was 1.78 (± 0.32), as shown in Table 1.

### 3.4. Comparison of Urodynamic Indicators of Patients

The maximum urine flow maximum detrusor systolic pressure and bladder compliance of patients before intervention were 9.67 (± 0.68) mL/s, 13.02 (± 1.02) cmH2O, and 25.98 (± 1.79) cmH2O, respectively, while those after intervention were 13.78 (± 1.29) mL/s, 19.78 (± 3.10) cmH2O, and 35.28 (± 3.20) cmH2O. The operative incisions of patients healed within 2 weeks without pressure ulcers, infection, and other complications. See Table 2.

### 3.5. Radiographic Comparison of Preoperative and Postoperative Outcomes

To further illustrate the effectiveness of the posterior decompression and internal fixation surgery, we included radiographic images showing the condition of the spine before and after the intervention.

**Figure 1:**
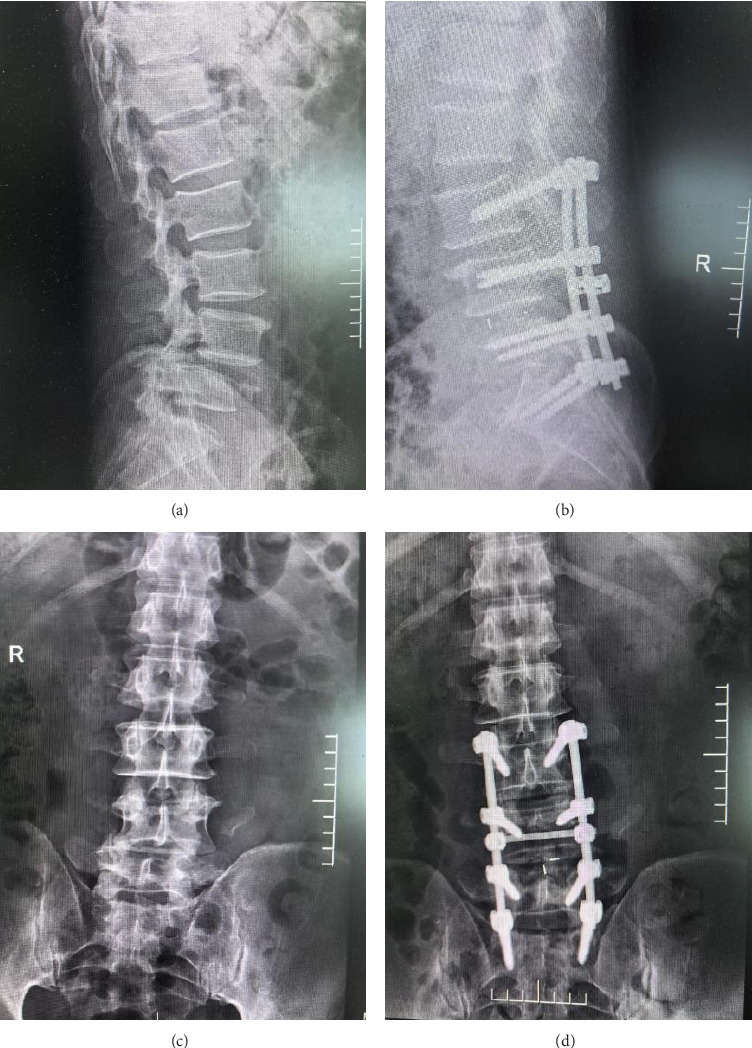
Radiographic images of the thoracolumbar spine in a patient with spinal cord injury before and after posterior decompression and internal fixation surgery: (a) preoperative lateral view; (b) postoperative lateral view; (c) preoperative anteroposterior view; (d) postoperative anteroposterior view.

Figure 1 demonstrates the preoperative and postoperative radiographs. Images A and C show the lateral and anteroposterior views of the spine prior to surgery, indicating the fracture and misalignment. Images B and D display the lateral and anteroposterior views after surgery, highlighting the placement of internal fixation devices and the corrected alignment of the spine. The preoperative images (Figures 1(a) and 1(c)) reveal significant vertebral body collapse and misalignment. Postoperatively (Figures 1(b) and 1(d)), the vertebral bodies are realigned, and the internal fixation devices are properly positioned, indicating a successful surgical outcome.

## 4. Discussion

Thoracolumbar fracture with spinal cord injury is common with high incidence among middle-aged and young people. With the rapid economic development, the incidence of such fracture is increasing year by year [8]. As the central pillar of the body, the lumbar spine with high mobility needs to support the weight of the body, playing an important role of load-bearing capacity. Traffic accidents and high falling can easily lead to thoracolumbar fractures. Bone fragments after a fracture will cause injury of the spinal canal to some extent, resulting in impairment of spinal nerve function, which may affect the prognosis activity of patients in severe cases [9, 10]. It is necessary to take prompt treatment to relieve the injury of the spinal cauda equina nerve caused by the fracture, and timely scientific and effective intervention measures have positive influence on the recovery of patients' prognostic function and improvement of prognostic quality of life.

Currently, surgical interventions are mainly used in the treatment of thoracolumbar fractures with spinal cord injury. Anterior decompression and fixation can completely relieve the compression of the compression material in front of the spine without aggravating the spinal cord injury and avoid the failure to maintain normal height of the vertebral body and the loss of the spinal kyphosis angle after surgery as much as possible. Although a few scholars suggest that a spinal fracture and incurred spinal cord injury are mainly affected by the impact force in front of the spinal cord, and it is necessary to focus on anterior or anterolateral decompression. However, it must be very familiar with the anatomical structure during the actual surgery, and a series of complications are likely to occur or potential to occur during the surgery. It is necessary to ensure the point of insertion and the direction of the screw to reduce or avoid the screw entering the spinal canal by mistake. Therefore, anterior decompression and internal fixation are no longer recommended. The intervention effect of posterior decompression and internal fixation for treatment of thoracolumbar fracture complicated with spinal cord injury still needs to be further discussed according to the actual situation of patients [11–13].

Most scholars suggest that patients with thoracic vertebral fracture complicated with spinal cord injury should be treated with surgical intervention for fracture reduction as soon as possible to relieve the compression of broken bones on surrounding tissues and nerve tissues, increase the spinal canal volume, alleviate or avoid secondary spinal cord injury as much as possible, and eliminate the metabolites produced by toxic reaction as soon as possible [14, 15]. The internal fixation for reduction and fixation can reconstruct the spinal structure and ensure the stability of the spine. However, there has been controversy about the surgical approach [16]. Posterior decompression and internal fixation can meet the treatment needs of patients and improve the anterior border height of the vertebral body, with an ideal effect. The anatomical structure of posterior decompression and internal fixation is relatively simple and less invasive; thus, the postoperative pain is relatively lower compared with that of other decompression and internal fixation therapies. Furthermore, it is safe and reliable since its postoperative complications are relatively less. The research data showed that the mean surgery time, mean intraoperative blood loss, and mean hospital stay of the 40 patients included in this study were 115.34 (± 7.38) min, 312.98 (± 40.37) mL, and 16.83 (± 0.48) d, respectively. The anterior border height of the injured vertebral body in patients treated with posterior decompression and internal fixation was significantly improved, with the anterior border height of the injured vertebral body recovered to 12.8 2(± 1.23) mm on average, the posterior border height of the injured vertebral body recovered to 3.21 (± 0.64) mm on average, and the angle of spinal kyphosis recovered to 14.26 (± 0.32)° on average. Alongside the observed improvements, it is crucial to consider the possible side effects associated with the surgical procedure, which include infection, nerve damage, and blood loss. To mitigate these risks, comprehensive preventive measures were implemented. Antibiotic prophylaxis was administered to all patients to prevent postoperative infections. Intraoperative nerve monitoring was conducted to avoid nerve damage during the surgical procedure. Moreover, meticulous surgical techniques were employed to minimize tissue trauma and ensure accurate hardware placement, thus reducing the likelihood of significant blood loss.

According to the data, the motor function score and sensory function score in the ASIA scale before surgery were 40.78 (± 4.32) and 45.98 (± 3.20), respectively, while those after surgery were 59.86 (± 1.37) and 66.92 (± 1.28). The VAS score of patients was 6.89 (± 0.78) before intervention, while that of patients after intervention was 1.78 (± 0.32). The maximum urine flow maximum detrusor systolic pressure and bladder compliance of patients before intervention were 9.67 (± 0.68) mL/s, 13.02 (± 1.02) cm H_2_O, and 25.98 (± 1.79) cm H_2_O, respectively, while those after intervention were 13.78 (± 1.29) mL/s, 19.78 (± 3.10) cm H_2_O, and 35.28 (± 3.20) cm H_2_O. The operative incisions of patients healed within 2 weeks without pressure ulcers, infection, and other complications. The analysis suggests that the posterior approach is more convenient and less invasive than other approaches, which can remove the fractured bone substances of the vertebral body and perform reduction after the lateral approach to the anterior position of the spinal canal, complete the decompression of the spinal canal with minimal trauma to prevent injury, and ensure the recovery of neurological function as soon as possible. The use of a pedicle screw system for posterior decompression and open reduction appears to be more effective in clinical interventions, without compression in front of the dural sac, which improves the motor function and sensation of the lower limb and alleviates the pain caused by trauma [17].

In discussing the implications of our findings, it is important to acknowledge the limitations in terms of sample representativeness. While our study sample included a diverse age range and both genders, the representativeness might be limited geographically and racially, as all participants were from the same hospital, which may limit the generalizability of the findings. Future studies should consider including more varied demographic settings to enhance generalizability. This will help to confirm whether our findings can be applied more broadly to different populations.

For future studies, it is recommended to include a multicenter design, incorporate control groups using alternative surgical techniques, and consider a broader demographic scope. Extending the follow-up period would also provide insights into the long-term efficacy and safety of the surgical interventions. Such studies could help confirm whether our findings are applicable more broadly across different settings and patient populations.

In summary, the findings of this study suggest that posterior decompression and internal fixation offer a promising intervention for patients suffering from thoracolumbar fractures complicated by spinal cord injuries. This technique has demonstrated potential to not only reduce surgical and intraoperative blood loss durations but also to significantly enhance the structural integrity of the spine by increasing the height of the anterior vertebral border. Furthermore, it contributes to considerable alleviations in pain levels and notable improvements in both motor and sensory functions of the lower limbs. Future research should ideally be conducted through larger scale, randomized controlled trials to more definitively ascertain the efficacy of this surgical approach across diverse clinical settings.

## Figures and Tables

**Table 1:** Comparison of ASIA score and VAS score of patients (score, $\bar{x}\pm s$).

Group	Number of cases	ASIA score		VAS score
		Motor function	Sensory function	Before intervention
Before intervention	40	40.78 ± 4.32	45.98 ± 3.20	6.89 ± 0.78
After intervention	40	59.86 ± 1.37	66.92 ± 1.28	1.78 ± 0.32
*t*	—	28.242	40.757	16.248
*p*	—	< 0.05	< 0.05	< 0.05

Abbreviations: ASIA, American Spinal Injury Association; VAS, Visual Analog Scale.

**Table 2:** Comparison of urodynamic indicator of patients ($\bar{x}\pm s$).

Group	Number of cases	Max. urine flow (mL/s)	Max. detrusor systolic pressure (cmH2O)	Bladder compliance (cmH2O)
Before intervention	40	9.67 ± 0.68	13.02 ± 1.02	25.98 ± 1.79
After intervention	40	13.78 ± 1.29	19.78 ± 3.10	35.28 ± 3.20
*t*	—	18.907	13.895	17.015
*p*	—	< 0.05	< 0.05	< 0.05

## Data Availability

The simulation experiment data used to support the findings of this study are available from the corresponding author upon request.
